# Long non‐coding RNA OIP5‐AS1 (Cyrano): A context‐specific regulator of normal and disease processes

**DOI:** 10.1002/ctm2.706

**Published:** 2022-01-18

**Authors:** Serena Wooten, Keriayn N. Smith

**Affiliations:** ^1^ Department of Genetics University of North Carolina at Chapel Hill North Carolina USA

**Keywords:** cancer, competing endogenous RNAs, Cyrano, diagnostic, disease, long non‐coding RNAs, miRNAs, OIP5‐AS1, Oip5os1, prognostic, therapeutics

## Abstract

Long non‐coding (lnc) RNAs have been implicated in a plethora of normal biological functions, and have also emerged as key molecules in various disease processes. OIP5‐AS1, also commonly known by the alias *Cyrano*, is a lncRNA that displays broad expression across multiple tissues, with significant enrichment in particular contexts including within the nervous system and skeletal muscle. Thus far, this multifaceted lncRNA has been found to have regulatory functions in normal cellular processes including cell proliferation and survival, as well as in the development and progression of a myriad disease states. These widespread effects on normal and disease states have been found to be mediated through context‐specific intermolecular interactions with dozens of miRNAs and proteins identified to date. This review explores recent studies to highlight OIP5‐AS1's contextual yet pleiotropic roles in normal homeostatic functions as well as disease oetiology and progression, which may influence its utility in the generation of future theranostics.

## Graphical abstract

Long noncoding RNAs have emerged as critical regulators of normal and disease processes. OIP5‐AS1, commonly known as Cyrano, has been remarkably associated with a myriad normal biological functions and numerous disease states, based on its far‐reaching functional complexity. This review explores OIP5‐AS1's role in both normal cellular functions and disease emergence, progression, and prospects for clinical utility.

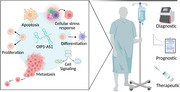

## 1 BACKGROUND

Long non‐coding RNAs (lncRNAs) are defined as RNAs that are more than 200 nucleotides in length, which are transcribed through mechanisms similar to mRNA and yet, lack significant protein‐coding potential.^1^ LncRNAs have been found to serve many functions in biological processes of human relevance, including the regulation of cell growth, proliferation and death; homeostasis; tissue/organ development; as well as regulating specialised or anomalous characteristics or processes such as pluripotency and self‐renewal, and the development and progression of certain diseases, respectively.^2^, ^3^, ^4^, ^5^, ^6^, ^7^

LncRNAs can be found in various locations throughout the genome relative to protein‐coding genes, including in overlapping and non‐overlapping positions (e.g. sense‐overlapping, antisense‐overlapping, intronic and intergenic).^8^, ^9^ In some cases, such as for some cis‐regulatory roles, their genomic location provides information on their function; however, for the majority of lncRNAs, genomic location provides little information on their roles in the cell.^8^, ^9^, ^10^

Some insight on the function of lncRNAs can be gleaned from their tissue, cellular and subcellular location, and a notable property of lncRNAs is their markedly specific cellular and subcellular localisation.^11^, ^12^, ^13^ Within these regions, lncRNAs use very diverse mechanisms including functioning as molecular decoys, guides, scaffolds, activators or inhibitors, to influence transcriptional, post‐transcriptional and translational processes. Through these mechanisms, they are increasingly recognised as regulators of cell fate, homeostasis, proliferation and cell survival in normal and disease conditions.^11^, ^14^, ^15^ These roles are functionally dictated by their intermolecular interactions including with other RNA molecules, chromatin and proteins (thoroughly reviewed in Refs.^1^, ^8^, ^16^, ^17^, ^18^, ^19^).

**FIGURE 1:**
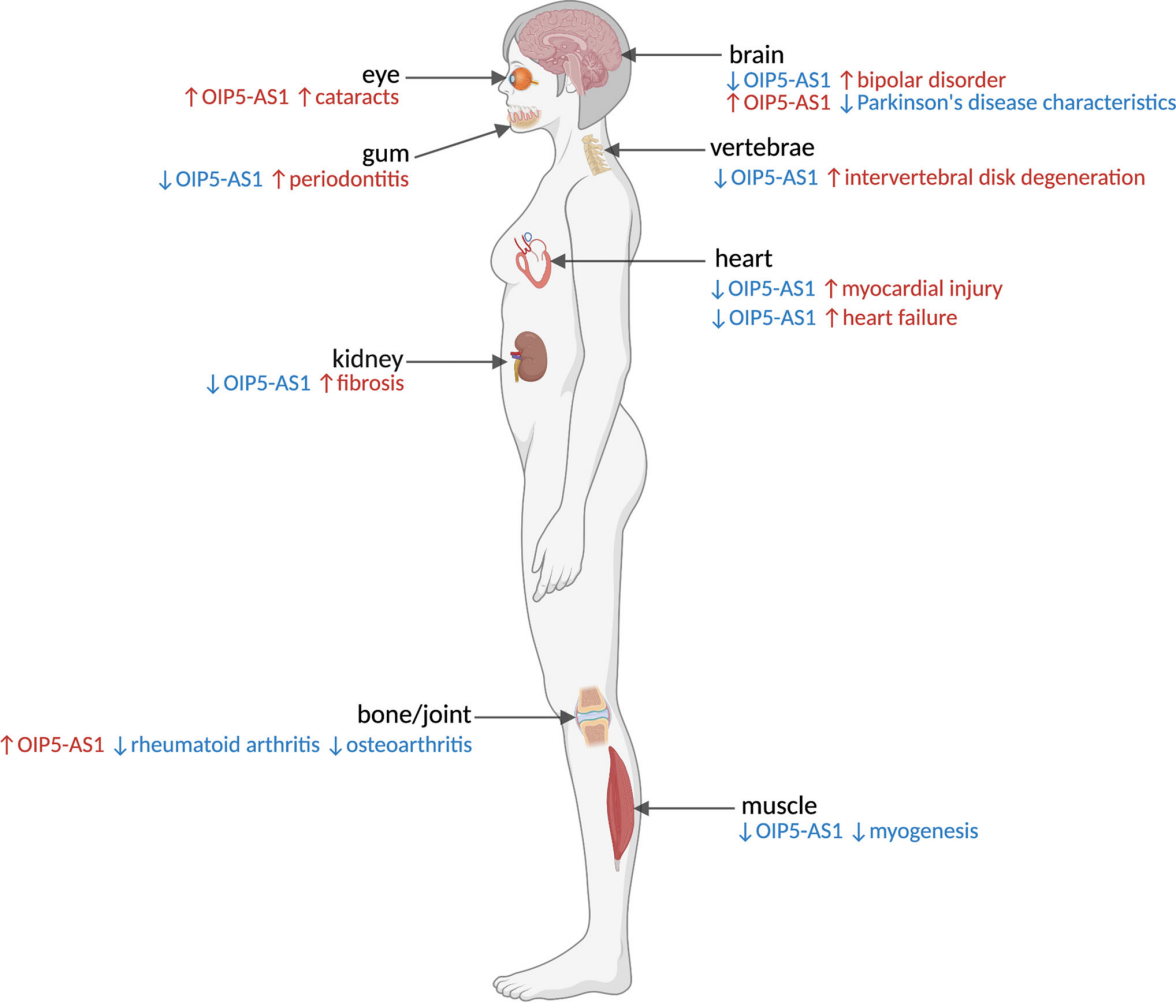
The single long non‐coding RNA (lncRNA) (Cyrano; OIP5‐AS1 in human; Oip5os1 or 1700020I14Rik in mouse) has been associated with normal and aberrant cellular processes in diseases occurring in various tissues/organs. Tissue colours serve only to provide contrast and tissues/organs are not drawn to scale, and are not an exclusive list

OIP5‐AS1 (human; *terminology used throughout for clarity*), also known as Oip5os1 (or 1700020I14Rik; mouse) or Cyrano (general), is often described as a canonical lncRNA. It has been implicated in developmental processes, as well as the regulation of a wide variety of cellular processes, including proliferation, apoptosis and mitosis.^1^, ^11^, ^13^, ^20^ Studies have also suggested roles for OIP5‐AS1 in several chronic conditions and illnesses (Figure 1; Table 1) as well as in the onset and progression of various cancers (Table 2).^2^, ^3^, ^4^, ^5^, ^6^, ^7^, ^21^, ^22^, ^23^, ^24^

**TABLE 1:** Cellular processes associated with OIP5‐AS1 function in human disease

Disorder	Cellular process	Mediators	Model	References
Acute lung injury	Proliferation, apoptosis, oxidative stress response, inflammatory response	miR‐223, NLRP3, miR‐26a‐5p, TLR4	Cell lines, animal models	^180^ ^,^ ^181^
Asthma	Apoptosis regulation, inflammatory response, miRNA regulation	miR‐143‐3p, HMGB1	Cell lines, clinical specimens	^79^, ^80^
Atherosclerosis	miRNA regulation, apoptosis regulation	miR‐320a, LOX1	Cell lines and clinical specimens	^98^
Bipolar disorder	Unknown, potential biomarker based on expression	Unknown	Cell lines and clinical specimens	^83^
Cataracts	Apoptosis, oxidative stress	HuR, POLG	Cell lines	^73^
Cardiac failure	Mitochondrial function, stress response in female animals	Unknown	Cell lines and animal models	^109^
Diabetic nephropathy	Apoptosis, proliferation, fibrosis	miR‐34a‐5p, Sirt1, HIF‐1α	Cell lines and animal models	^54^
Diabetic angiopathy	Apoptosis, cognitive function	miR‐200b, ACE2	Cell lines and animal models	^57^
Diabetic retinopathy	Apoptosis	miR‐449c	Cell lines and clinical specimens	^59^
Intervertebral disk degeneration	Proliferation, extracellular matrix accumulation	Unknown	Cell lines and clinical specimens	^72^
Myocardial ischaemia	Apoptosis	miR‐297a, CGRP, miR‐29a, SIRT1	Cell lines and animal models	^2^, ^62^
Osteoarthritis	Inflammatory response regulation, apoptosis, migration, proliferation	miR‐30a‐5p, miR‐29b‐3p, PGRN	Cell lines and clinical specimens	^66^, ^182^
Periodontitis	Inflammatory response	Unknown	Cell lines and clinical specimens	^76^
Parkinson's disease	Alpha‐synuclein toxicity and aggregation, apoptosis, autophagy, endoplasmic reticulum stress	miR‐126	Cell lines and clinical specimens	^92^
Rheumatoid arthritis	Inflammatory response regulation, apoptosis	miR‐448, PON1	Cell lines and clinical specimens	^65^
Schizophrenia	Disease predictor in females	Unknown	Cell lines and clinical specimens	^22^

Abbreviations: CGRP, calcitonin gene‐related peptide; LOX1, lectin‐like oxidised low‐density lipoprotein 1; PGRN, progranulin; PON1, paraoxonase 1; Sirt1, silent information regulator T1.

**TABLE 2:** Roles for OIP5‐AS1 in cancers

Cancer	Process	Proposed role	Mediators	Model	References
Bladder cancer	Cell proliferation, apoptosis	Oncogene	miR‐217, MTDH	Cells and clinical specimens	^118^, ^133^, ^134^
Brain cancer	Cell proliferation, survival/apoptosis, migration, invasion, tumour growth, temozolomide resistance	Oncogene tumour suppressor	miR‐367‐3p, PIWIL3, YAP, notch signalling, miR‐410, Wnt pathway, POX, miR‐129‐5p, IGF2BP2	Cells and clinical specimens	^135^, ^136^, ^137^, ^138^, ^139^
Breast cancer	Cell proliferation, migration, invasion, apoptosis, angiogenesis	Oncogene	miR‐129‐5p, Sox2, YAP1, JAG1, miR‐216a‐5p, GLO1, miR‐340‐5p, ZEB2	Cells, clinical specimens, and mouse studies	^140^, ^141^, ^142^, ^143^
Cervical cancer	Cell proliferation and cell invasion	Oncogene	miR‐143‐3p, ITGA6, SMAD3, ROCK1	Cells and clinical specimens	^144^, ^145^, ^146^
Colorectal cancer	Proliferation, migration, invasion, apoptosis, radioresistance, oxaliplatin resistance	Oncogene	miRNA‐137, miR‐369‐3p, DYRK1A, miR‐34b‐5p, HuR	Cells and clinical specimens	^24^, ^147^, ^148^, ^149^
Endometrial cancer	Cell proliferation, migration, invasion, EMT	Oncogene tumour suppressor	miR‐152‐3p, SLC7A5, miR‐200c‐3p, PTEN	Cells, clinical specimens, and mouse studies	^150^, ^151^
Oesophageal cancer	Cell proliferation, migration, invasion	Oncogene	miR‐30a, VOPP1	Cells and clinical specimens	^152^
Gallbladder cancer	Cell migration and invasion	Oncogene	miR‐143‐3p	Cell lines	^153^
Gastric cancer	Cell proliferation, migration, invasion, apoptosis	Oncogene	EZH2, NLRP6, miR‐367‐3p, HMGA2, miR‐153‐3p, ZBTB2, miR‐186	Cells, clinical specimens and mouse studies	^49^, ^154^, ^155^, ^156^
Liver cancer	Cell proliferation, apoptosis, EMT, migration, invasion, angiogenesis	Oncogene	miR‐186a‐5p, Zeb1, miR‐26a‐3p, EPHA2, miR‐363‐3p, SOX4, miR‐3163, VEGFA, miR‐300, YY1	Cells, clinical specimens and mouse studies	^51^, ^157^, ^158^, ^159^, ^160^
Lung cancer	Cell proliferation, migration, invasion	Oncogene	miR‐448, BCL‐2, miR‐378a‐3p, CDK4, CDK6, miR‐140‐5p, HDAC7, VEGFA	Cells, clinical specimens and mouse studies	^161^, ^162^, ^163^
Multiple myeloma	Regulates cell proliferation and apoptosis	Tumour suppressor	miR‐410, KLF10/PTEN/AKT pathway, miR‐27a‐3p, TSC1	Cells, clinical specimens and mouse studies	^7^, ^129^
Nasopharyngeal cancer	Cell proliferation, migration, invasion, apoptosis	Oncogene	miR‐203	Cells, clinical specimens and mouse studies	^164^
Oral cancer	Associated with stemness signature, cell proliferation, migration, invasion, cisplatin resistance	Oncogene	Various miRNAs, NRP1, TRIM14	Cells, clinical specimens and mouse studies	^165^, ^166^, ^167^
Osteosarcoma	Regulates cell proliferation and apoptosis, angiogenesis, autophagy, cisplatin resistance, doxorubicin resistance	Oncogene	miR‐340‐5p, PI3K/AKT/mTOR pathway, miR‐200b‐3p, fibronectin, miR‐223, CDK14, miR‐137, pleiotrophin (PTN), miR‐377‐3p, miR‐153, ATG5	Cells, clinical specimens and mouse studies	^122^, ^123^, ^124^, ^125^, ^168^, ^169^
Ovarian cancer	Cell proliferation, glycolysis, apoptosis, EMT, migration and invasion	Oncogene	miR‐34a, Snai1, miR‐137, ZNF217, miR‐324‐3p, NFIB, miR‐128‐3p, CCNG1	Cells, clinical specimens and mouse studies	^170^, ^171^, ^172^, ^173^
Pancreatic cancer	Cell proliferation, EMT, migration, metastasis	Oncogene	miR‐429, FOXD1, miR‐342‐3p, AKT/ERK pathway, miR‐186‐5p	Cells, clinical specimens and mouse studies	^174^, ^175^, ^176^
Skin cancer	Cellular metabolism	Oncogene	miR‐217, glutaminase (GLS)	Cells and clinical specimens	^177^
Thyroid cancer	Cell proliferation, migration	Oncogene	FXR1, YY1, CTNNB1, miR‐98, ASAMTS8, EGFR signalling, MEK/ERK signalling	Cells and clinical specimens	^178^, ^179^

Abbreviation: EMT, epithelial‐mesenchymal transition.

We provide a synopsis of the molecular mechanisms which mediate OIP5‐AS1‐associated functions in normal and disease contexts through description of its pleiotropic expression and intermolecular interactions. As such, this review serves as a framework for how a single‐multifunctional lncRNA holds context‐specific roles, and discusses the implication of this pleiotropism in human disease, with a view towards how this can affect the outlook on diagnoses, prognoses and therapies.

## 2 REGULATORY ROLE OF OIP5‐AS1 IN CELLULAR FUNCTIONS

### 2.1 Sequence and structure conservation: Relation to function

Similar to mRNAs, many lncRNAs, including OIP5‐AS1, exhibit hallmarks of polyadenylation and capping, and likely depend on specific folding and structures to execute their functions.^25^ It is expected that sequence and structural properties mediate the recruitment and connection of interacting molecules for the regulation of processes such as transcription and translation.^1^, ^26^, ^27^

**HIGHLIGHTS**

1. OIP5‐AS1 (aliases: Oip5os1, 1700020I14Rik, Cyrano) is a pleiotropic lncRNA molecule.
2. OIP5‐AS1 has a vast array of functions in a myriad normal and disease processes, including neurological disorders, cancers and inflammatory disorders.
3. Based on this multifunctionality, OIP5‐AS1 has potential in translational medicine, including use as a diagnostic, prognostic or therapeutic agent, which requires clinical study.

OIP5‐AS1, whose most highly expressed isoform is more than 8 kb in length, exhibits minimal sequence conservation across vertebrate species from fish to mammals (including humans and mice), except for a highly conserved region of about 300–500 nucleotides in length.^20^ The functions of this region have been debated, with some studies pointing to developmental roles during zebrafish embryogenesis and proper cerebellar neuronal function, possibly based on the conserved structure.^20^, ^25^ However, the majority of functions of OIP5‐AS1 has been ascribed to intermolecular interactions with other molecules (Tables 1 and 2), with prospective binding sites located outside the conserved region.^5^ As indicated by the length of the transcript, there is significant potential for numerous interactions with chromatin, other RNAs, and proteins.^5^

### 2.2 Regulation of miR‐7 in neuronal activity

Early studies on OIP5‐AS1 focused on developmental contexts,^20^ and uncovered a unique relationship with the miRNA, miR‐7, with which it harbours an extensive interaction site within its highly conserved region.^4^, ^20^

Historically, miRNA function has primarily been ascribed to target RNA degradation and the inhibition of translation.^28^, ^29^ Conversely, one mechanism for the destabilisation of miRNAs is target‐directed miRNA degradation (TDMD).^4^, ^30^, ^31^ Here, OIP5‐AS1 has been found to be a potent mediator of miRNA destruction.^4^, ^31^ Specifically, through the highly complementary binding site, OIP5‐AS1 induces potent degradation of miR‐7, with miR‐7 levels markedly increasing upon its depletion.^4^ The outcome of this is the accumulation of the conserved circular RNA Cdr1as, which itself is negatively regulated by miR‐7.^4^, ^32^ This regulatory loop functions in the hippocampus and hypothalamus to modulate neuronal activity.^4^

### 2.3 Myogenesis

Myogenesis is a critical process in embryonic development and during muscle repair after injury.^33^, ^34^ Using human cell line models to replicate early stages of myogenesis, Yang et al.^23^ found that OIP5‐AS1's expression increases during myoblast development, and subsequently remains at high levels. Here, OIP5‐AS1 has broad functionality in muscle development and homeostasis, and its knockdown resulted in overall myogenesis suppression, reduced numbers and size of myotubes from myoblasts, large reduction in nuclei numbers, and a critical reduction in the activity of the differentiation indicator creatine kinase.^23^ Authors proposed a scaffolding function for OIP5‐AS1, where it supports HuR‐dependent stability of the myogenic transcription factor MEF2C mRNA.^23^

### 2.4 Defense against chemical stressors in HepG2 cells

The involvement of lncRNAs in chemical stress response has been poorly understood. Roles for OIP5‐AS1, FLJ46906, LINC01137 and GABPB1‐AS1 were examined using HepG2 cells as an in vitro model of the human detoxification process for chemicals, based upon exposure to different forms of chemical stress: hydrogen peroxide‐induced oxidative stress, mercury II oxide‐heavy metal stress and etoposide‐induced DNA damage.^6^

OIP5‐AS1 and the other three lncRNAs displayed differential expression due to the lengthening of the half‐lives of each lncRNA, with slower rates of decay in response to exposure to the cellular stressors.^6^ For OIP5‐AS1, authors proposed that higher levels provided a defense against chemical stress, based on its capacity to interact with various miRNAs and RNA‐binding proteins to regulate mitotic progression and cellular proliferation.^3^, ^6^, ^21^

### 2.5 Embryonic stem cell self‐renewal

Embryonic stem cells (ESCs) are rare self‐renewing, pluripotent cells that harbour a unique cell cycle profile that supports these properties.^35^ Their characteristics drive their utility in developmental biology studies and in regenerative medicine. LncRNAs are abundantly expressed in ESCs where they are involved in transcriptional and post‐transcriptional processes to support the maintenance of ESC characteristics.^36^, ^37^, ^38^, ^39^, ^40^

OIP5‐AS1 has been implicated in ESC maintenance, however recent evidence suggests that this may depend on the pluripotent cell type.^41^, ^42^ Its role in self‐renewal maintenance is postulated to at least be partially dependent on intermolecular interactions including with STAT3 and/or miR‐7.^5^ STAT3 is a transcription factor in the LIF/STAT3 signalling pathway that is necessary for the maintenance of mouse ESC self‐renewal and pluripotency,^43^, ^44^ that has emerged as a binding protein for lncRNAs.^5^, ^45^, ^46^

The interaction between STAT3 and OIP5‐AS1 is a possible mechanism for supporting the levels of the master pluripotency regulator, Nanog,^5^, ^42^ which is a downstream factor within the LIF/STAT3 signal pathway.^5^, ^42^, ^47^, ^48^ Levels of Nanog in mouse ESCs correlate with levels of OIP5‐AS1 within the cell, and if OIP5‐AS1 is silenced within the cell, then Nanog has also been found to be reduced as well. In addition to protection from a weak miR‐7 binding site, OIP5‐AS1's interaction with STAT3 to support maintenance of Nanog levels within the cell, is a mechanism to support ESC maintenance and self‐renewal.^5^, ^42^

### 2.6 Mitosis

Context specificity for OIP5‐AS1's function is also applicable for cell proliferation where it has an anti‐proliferative role in some contexts, and supports proliferation in others;^3^, ^49^, ^50^, ^51^ these roles possibly impact its functions in specific malignant conditions.^3^ For instance, the silencing of OIP5‐AS1 leads to the appearance of abnormal mitotic spindles, suggesting it is a key factor in mitotic progression within the cell.^3^ This function is mediated by interactions with multiple mRNAs, one example being the GAK mRNA, which is associated with cyclin G, and is important for cellular transition into and through metaphase.^3^

Both OIP5‐AS1 and GAK mRNA contain complementary regions to facilitate their interaction, and OIP5‐AS1 reduces the stability of GAK mRNA, thus reducing the amount of GAK protein present.^3^ Intriguingly, the outcome of OIP5‐AS1's negative regulation of GAK mRNA is proper chromosome alignment, separation and organisation during the metaphase of mitosis, with the silencing or knockdown of OIP5‐AS1 leading to disorganisation of chromosomes during metaphase.^3^ The importance of OIP5‐AS1 in successful mitosis was confirmed by partial rescue of normal mitotic spindle formation upon concurrent silencing of GAK.^3^ The outcome of OIP5‐AS1's role in mitosis likely extends to various diseases wherein cellular division is a critical component in the oetiology.

## 3 A SINGLE lncRNA: A MYRIAD DISEASES

### 3.1 Diabetes

**FIGURE 2:**
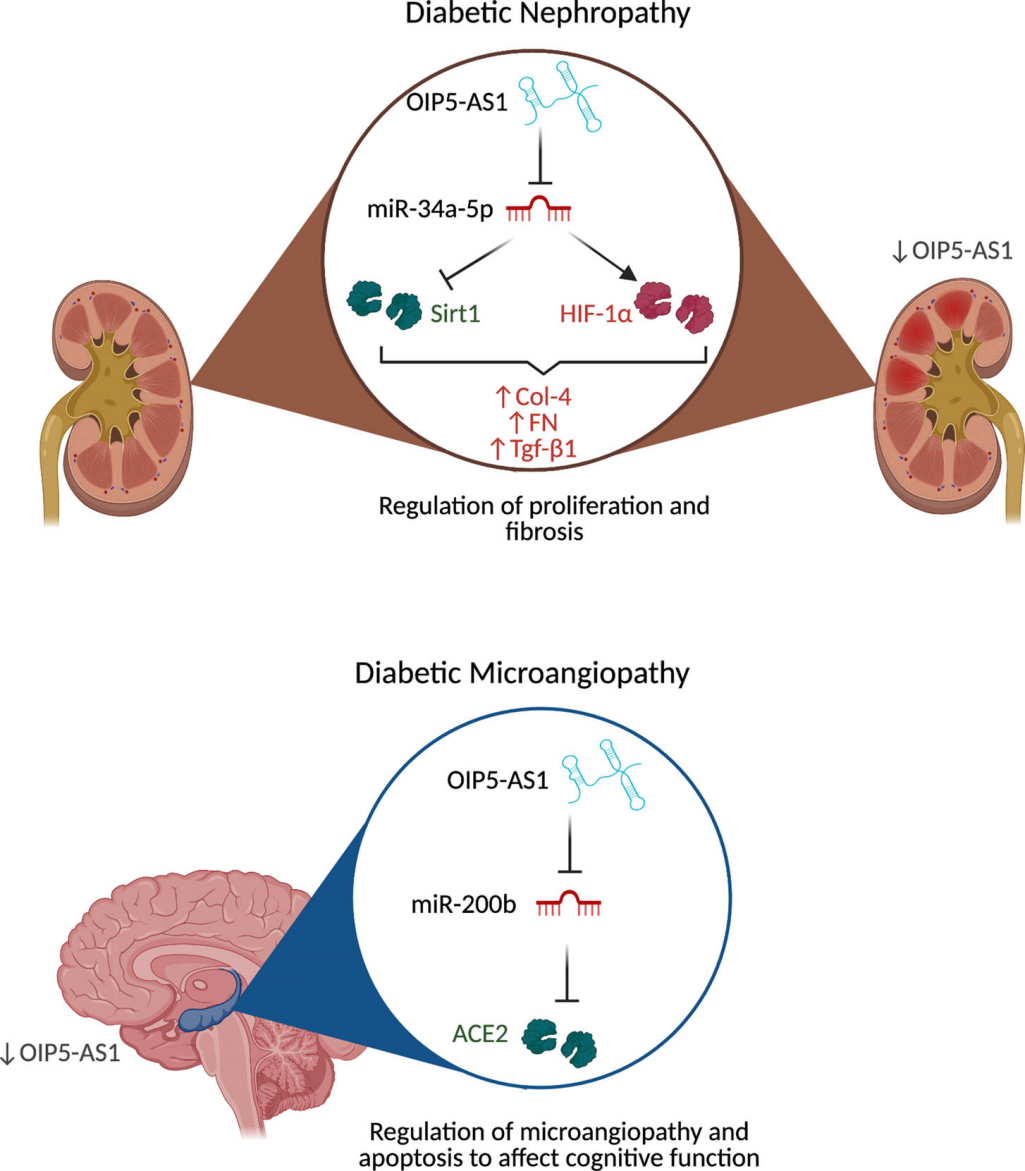
OIP5‐AS1 has been implicated in both nephropathy and microangiopathy associated with diabetes, based on its interactions with miRNAs. In diabetic nephropathy, its interactions with miR‐34a‐5p results in downstream effects on silent information regulator T1 (Sirt1), which is associated with fibrosis. In diabetic microangiopathy, effects are seen in the hippocampus, which results from OIP5‐AS1's interactions with miR‐200b to regulate ACE2

Diabetes mellitus is an increasingly prevalent chronic metabolic disorder with serious multisystem complications including microvascular pathologies such as nephropathy, neuropathy and retinopathy, in addition to macrovascular endpoints which include ischemic heart disease, peripheral vascular disease and stroke.^52^ OIP5‐AS1 has been found to be a competing endogenous RNA (ceRNA) in diabetic nephropathy (Figure 2), a main cause of end‐stage renal disease in diabetic patients.^53^, ^54^ This is based on its binding and regulation of miRNAs such as miR‐34a‐5p, and its functions in specific signalling pathways, both of which influence silent information regulator T1 (Sirt1) expression in renal tissues.^54^

miR‐34a‐5p is highly expressed in renal tissues in a high‐glucose environment.^55^ When expressed in renal cells, OIP5‐AS1 interacts with miR‐34a‐5p to release Sirt1 expression. As expression of Sirt1 increases, the reversal of fibrosis and the effects of inflammation—both key characteristics of diabetic neuropathy—can be observed in correlation.^54^, ^55^ Knockdown of OIP5‐AS1 resulted in increased cell proliferation in renal tissue cells, and its role in the progression to fibrosis has been studied by monitoring renal fibrosis‐related factors (collagen IV (Col‐4), fibronectin (FN), transcriptional regulatory factor‐beta 1 (TGF‐β1)) in relation to the levels of OIP5‐AS1 in renal tissue. This confirmed an inverse relationship between the levels of OIP5‐AS1 and renal fibrosis factors. Altogether, this suggests dual roles for OIP5‐AS1 in proliferation and fibrosis.

OIP5‐AS1 has also been associated with the pathogenesis of diabetic microangiopathy (Figure 2), a condition characteristic of diabetes that possibly reduces cognitive ability.^56^ OIP5‐AS1 expression was found to be lower in the hippocampus of diabetic mice compared to those of healthy specimens, and knockdown promotes microangiopathy in diabetic mice.^57^ This was due to the upregulation of miR‐200b and downstream reduction of angiotensin‐converting enzyme 2 (ACE2) in the hippocampus, which resulted in differential escape latency in Morris water maze tests.^57^ These data support a role for OIP5‐AS1 in microangiopathy and cognition in the context of diabetes.

Diabetic retinopathy is characterised by retinal endothelial cell (REC) dysfunction,^58^ yet the cause of abnormal REC cell function due to hyperglycaemia is not completely clear. In conditions of hyperglycaemia, OIP5‐AS1 was one of the most significantly downregulated genes, along with MYC, where authors proposed the existence of a ceRNA network centred around miR‐449c.^59^ In this network, OIP5‐AS1 was regulated by miR‐449c in human REC cells, supporting a hypothesis that this network plays a role in diabetic retinopathy.^59^

### 3.2 Myocardial ischaemia

Myocardial ischaemia is a leading cause of death worldwide,^60^ and it is also a prominent comorbidity with various disease states. One main treatment for myocardial ischaemia is reperfusion (blood flow restoration),^61^ which can lead to myocardial ischaemia reperfusion (I/R) injury due to myocardial cell and tissue damage.^61^

A recent study found that OIP5‐AS1 along with miR‐297a and calcitonin gene‐related peptide (CGRP) (OIP5‐AS1/miR‐297a/CGRP axis) has a critical role in regulating apoptosis in myocardial cells undergoing myocardial I/R injury.^2^ CGRP and miR‐297a expression was found to be negatively correlated after myocardial I/R injury, while the expression of CGRP positively correlated with the expression of OIP5‐AS1.^2^ Both OIP5‐AS1 (1700020I14Rik) and CGRP were downregulated in a myocardial I/R injury and hypoxia/reoxygenation injury model, while miR‐297a was upregulated and could also negatively regulate both OIP5‐AS1 and CGRP reporter constructs.^2^ The overexpression of OIP5‐AS1 triggered the upregulation of CGRP, reduced apoptosis and increased cell viability, whereas miR‐297a reversed the effects of OIP5‐AS1.^2^

Another study corroborated the findings of decreased expression of OIP5‐AS1 in rat hearts with myocardial I/R injury and a model of oxygen‐glucose deprivation/reoxygenation.^62^ In this instance, OIP5‐AS1's function was mediated through miR‐29a, where it functioned as a ceRNA to decrease its expression and upregulate SIRT1 expression. This action on the SIRT1/AMPK/PGC1α pathway served to attenuate the mitochondria‐mediated apoptosis induced by myocardial I/R.^62^

### 3.3 Rheumatoid arthritis and osteoarthritis

**FIGURE 3:**
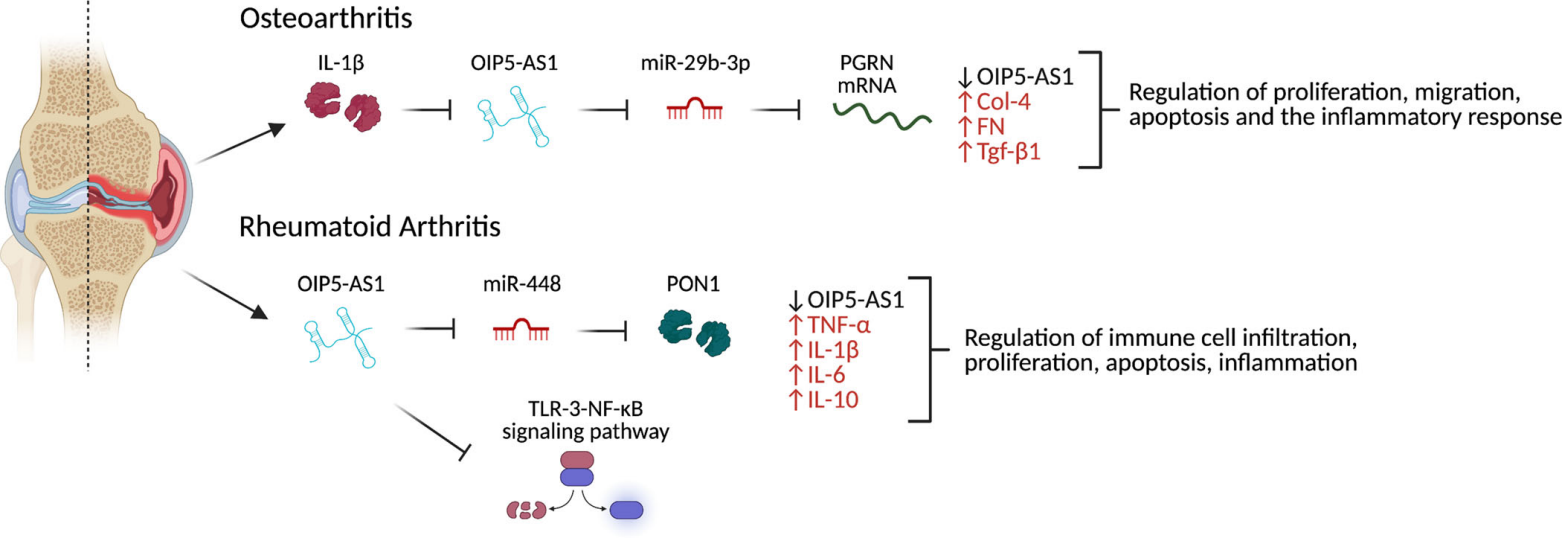
OIP5‐AS1 regulates inflammatory processes associated with both rheumatoid and osteoarthritis. In rheumatoid arthritis, it interacts with miR‐448 to regulate paraoxonase 1 (PON1), and is implicated in apoptosis and cell proliferation regulation. Its actions in osteoarthritis are mediated by miR‐29b‐3p to regulate apoptosis, inflammation, cell proliferation and migration

LncRNAs have been associated with the development and progression of rheumatoid arthritis through roles associated with inflammation and autoimmune regulation.^63^, ^64^ Inflammatory processes are common among rheumatoid and osteoarthritis, and OIP5‐AS1 has been found to be associated with inflammatory processes in both conditions (Figure 3).^65^, ^66^

Expression analysis in rats with rheumatoid arthritis implicated OIP5‐AS1 for further study, which found that its overexpression led to the reversal of certain aspects of rheumatoid arthritis. This included reduction in swelling, the number of immune cells present in the afflicted synovial tissue, and the levels of pro‐inflammatory cytokines (TNF‐α, IL‐1β, IL‐6 and IL‐10) expressed by synovial cells of rat models. These metrics suggest overall reduced rheumatoid arthritis severity.^65^

OIP5‐AS1's mechanism of action in rheumatoid arthritis is based on interactions with miR‐448, whereby it indirectly impacts levels of paraoxonase 1 (PON1), a miR‐448 target mRNA.^65^ Downregulation of OIP5‐AS1, the resulting increase in miR‐448 and correlated decrease in PON1 in synovial tissues, contributes to the inflammatory symptoms of rheumatoid arthritis. In addition to reducing symptom severity, immune cell infiltration and inflammation in animal models, OIP5‐AS1 was also implicated in cell proliferation and apoptosis regulation. Overexpression of OIP5‐AS1‐induced apoptosis and caused a decrease in the proliferation of fibroblast‐like synoviocyte (FLS) cells obtained from rheumatoid arthritis patients.^65^

Inflammation is also a hallmark of osteoarthritis,^67^ and the inflammatory cytokine IL‐1β inhibited OIP5‐AS1 expression in osteoarthritic tissues.^66^ This downregulation of OIP5‐AS1, concomitant with a decrease in progranulin (PGRN) mRNA was counter to that observed for miR‐29b‐3p, suggesting an antagonistic relationship.^66^ IL‐1β signals the release of other inflammatory cytokines (IL‐6, IL‐8 and TNF‐α), and the decrease of OIP5‐AS1 in CHON‐001 and ATDC5 cell lines triggers the upregulation of miR‐29b‐3p, for which it contains a binding sequence, which in turn resulted in apoptosis and an inflammatory response. In contrast, the upregulation of OIP5‐AS1 in these cell lines reversed these effects, ameliorating apoptosis and inflammation, and supported increased proliferation and cellular migration.^66^

Together, these data suggest that OIP5‐AS1 is relevant in the study of both osteoarthritis and rheumatoid arthritis, based on its regulatory roles in proliferation, migration, apoptosis and inflammation.

### 3.4 Intervertebral disk degeneration

The dysregulation of lncRNA expression influences apoptosis of disk cells, implying relevance in the development of intervertebral disk degeneration (IDD).^68^, ^69^ OIP5‐AS1 has specifically been implicated in IDD where it has been found in a ceRNA network (along with KCNQ10T1 and UGDH‐AS1) to regulate hsa‐miR‐140, which is involved in cartilage development and osteoarthritis pathogenesis.^70^ The stimulation of cell proliferation and extracellular matrix accumulation could slow or even reverse degenerative conditions such as IDD progression.^70^, ^71^ Based on its action as a sponge that prevents the HuR protein from binding to its target mRNA, authors hypothesised that OIP5‐AS1's role in IDD could be through regulation of cellular proliferation, or via regulating the expression of FOXF1 and PKD1.^21^, ^72^ However, further studies are needed on the precise mechanisms behind the association of OIP5‐AS1 with IDD.

### 3.5 Cataracts

OIP5‐AS1 has been implicated in the development of cataracts, where under oxidative stress, it is activated by TFAP2A and upregulated in B3 cells and lens epithelial cells.^73^ Knockdown of OIP5‐AS1 resulted in a reduction of B3 cell apoptosis due to H_2_O_2_‐induced oxidative stress, as well as an absence of lens opacity under oxidative stress, all of which contribute to the development of cataracts.^73^ The hypothesis is that OIP5‐AS1 contributes to regulation of the HuR‐mediated mitochondrial apoptosis pathway in B3 cells by mediating the destruction of POLG mRNA in the presence of oxidative stress, which could support the eventual development of cataracts.^73^

### 3.6 Periodontitis

Periodontitis has hallmarks associated with inflammatory diseases, as it is marked by chronic inflammation of the periodontium.^74^ Studies have attempted to determine the molecular mechanisms behind the manifestation of periodontitis via lncRNAs that are known to regulate inflammatory processes. OIP5‐AS1 is one such lncRNA, where its levels are reduced in tissues with periodontitis compared to healthy tissues.^75^, ^76^ It has also been found that expression of OIP5‐AS1 is significantly lower in female periodontitis patients relative to their male counterparts.^76^ It is hypothesised that the downregulation of OIP5‐AS1 allows for a subsequent rise in HuR activity, which stimulates the inflammatory responses that result in periodontitis.^76^

### 3.7 Asthma

Previous studies have been done to determine whether lncRNAs can be used as biomarkers for chronic respiratory diseases such as asthma and chronic obstructive pulmonary disease (COPD).^77^, ^78^ OIP5‐AS1 shows differential expression in models of asthma relative to controls,^79^, ^80^ and as such, it has been found to have discriminative power as a potential biomarker for asthma.^77^, ^79^ Its functions in apoptosis regulation and inflammatory processes in multiple cell types, and the association of these characteristics with bronchial asthma, also justified studies of OIP5‐AS1 in asthma.

Using a human bronchial epithelial cell model, Cai et al.^79^ observed that depletion of OIP5‐AS1 reduced apoptosis and inflammatory cytokine expression (TNF‐α, IL‐8). Additionally, OIP5‐AS1 interacts with, and negatively regulates miR‐143‐3p, to increase inflammatory response and apoptosis, illustrating potential for regulating processes that contribute to the pathogenesis of asthma.^79^ These data highlight the pleiotropic effect of OIP5‐AS1 in varying biological contexts.

### 3.8 Schizophrenia

Schizophrenia has been recognised as a disabling, genetic mental disorder.^81^ Various lncRNAs are dysregulated in cases of schizophrenia, and lncRNAs therefore show promise as biomarkers for schizophrenia diagnosis.^82^ However, their roles in the onset and progression of schizophrenia have not been fully explored and remains a relatively novel subject.

Recent studies have been carried out on OIP5‐AS1 along with other lncRNAs (FAS‐AS1, PVT1, TUG1, THRIL, NEAT1 and GAS5) due to their involvement in neurodevelopmental and/or neurobiological processes, and their involvement in signalling pathways present in patients with schizophrenia. Levels of GAS5, NEAT1 and OIP5‐AS1 were generally similar between patients diagnosed with schizophrenia and those negative for schizophrenia.^22^ However, when comparing data between sexes (males vs. females), GAS5, NEAT1 and OIP5‐AS1 expression levels had a higher and more significant association with schizophrenia in female patients compared to males.^82^

Studies also showed a correlation in expression between lncRNAs PVT1 and OIP5‐AS1; PVT1 and OIP5‐AS1 expression levels were shown to be directly correlated as well as sex dependent.^22^, ^82^ In the process of diagnosing schizophrenia, OIP5‐AS1 displayed a sensitivity level of 100% and a specificity level of 60.78% in diagnosing female patients with schizophrenia. OIP5‐AS1 levels also displayed a significant increase in female patients under the age of 50 years old.^22^ This suggests utility as a diagnostic tool for schizophrenia.

### 3.9 Bipolar disorder

LncRNAs have also been linked to bipolar disorder, where the expression levels of apoptosis‐related lncRNAs were assessed using peripheral blood of patients.^83^ Among them, OIP5‐AS1 expression was found to be significantly downregulated in patients with bipolar disorder. Along with the assessment of the expression of CCAT2, TUG1 and PANDA transcription levels, OIP5‐AS1 helped to improve the sensitivity and specificity for bipolar disorder diagnosis to 96%, and is thus a potential biomarker. Further studies are needed to explore its role in disease pathogenesis, as well as how lncRNAs such as OIP5‐AS1 could potentially provide a template for improved treatment in patients with bipolar disorder.

### 3.10 Parkinson's disease

Based on the complexities of Parkinson's disease, significant effort is underway to unravel the mechanism behind the characteristic dopaminergic neuron loss.^84^, ^85^ Methods such as immunotherapy are being used to treat underlying symptoms of Parkinson's disease such as alpha‐synuclein aggregation and toxicity.^86^

Similar to other neurological disorders, lncRNA action has also been associated with Parkinson's disease.^87^, ^88^, ^89^, ^90^, ^91^ For OIP5‐AS1, it was found to impede characteristics associated with Parkinson's disease, as it reduced alpha‐synuclein aggregation and toxicity via miR‐126 binding, yielding reduced rates of apoptosis and neuron loss.^92^ Another mechanism through which OIP5‐AS1 is involved is through the regulation of autophagy by inhibiting endoplasmic reticulum stress.^92^ These multiple mechanisms suggest that OIP5‐AS1 may have multifaceted roles in Parkinson's disease.

### 3.11 Atherosclerosis

Atherosclerosis can be characterised by the buildup of lipids and fibrous elements in medium and large arteries, which can lead to a variety of other cardiovascular ailments and chronic conditions such as heart disease, peripheral vascular disease and cerebral infarction.^93^, ^94^, ^95^ As endothelial cells are critical for maintaining vascular homeostasis, endothelial dysfunction is a hallmark of atherosclerosis.^96^ Therefore, monitoring characteristics such as endothelial cell proliferation, metabolism and apoptosis could provide a framework for atherosclerosis therapy and prevention.

**FIGURE 4:**
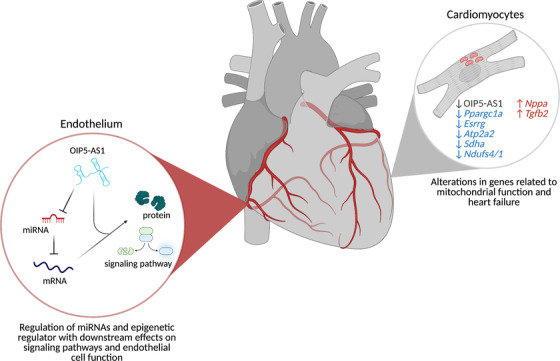
OIP5‐AS1's diverse mechanisms of action influence cardiovascular health. OIP5‐AS1's depletion is associated with cardiac failure in female animals, where it affects factors involved in the regulation of cellular metabolism. In atherosclerosis, its interactions with miRNAs (miR‐320a, miR‐26a) and the epigenetic regulator EZH2 mediate endothelial cell function via proteins such as lectin‐like oxidised low‐density lipoprotein 1 (LOX1) and signalling pathways such as GSK3β, AKT and NF‐κβ

Studies have suggested a role for OIP5‐AS1 in endothelial cell function (Figure 4).^97^, ^98^, ^99^, ^100^ This includes computational studies that identify OIP5‐AS1 in ceRNA networks that are proposed to have regulatory function in atherosclerosis‐related cellular processes.^101^ Additionally, studies used human umbilical vein endothelial cells (HUVEC) treated with oxidative low‐density lipoprotein (ox‐LDL) as a model to investigate mechanisms for atherosclerosis development. Here, a pathway involving OIP5‐AS1's interaction with miR‐320a and the downstream effects on lectin‐like oxidised low‐density lipoprotein 1 (LOX1) is associated with atherosclerosis characteristics. OIP5‐AS1 was increased in ox‐LDL‐treated HUVEC and its knockdown attenuated the effects on cell viability and apoptosis that were induced by ox‐LDL.^98^

miR‐320a has been found to be involved with atherogenesis,^102^ and it was identified as an interacting partner of OIP5‐AS1, where it exists in a negative feedback relationship.^98^ OIP5‐AS1 directly downregulates miR‐320a, while miR‐320a can also act as a negative regulator of OIP5‐AS1.^98^ LOX1 is a poorly understood scavenger protein, implicated in atherosclerosis development by inducing uptake of ox‐LDL by endothelial cells.^103^ Increased expression of LOX1 contributes to vascular cell injury since it inhibits endothelial cell growth and proliferation.^98^, ^103^ OIP5‐AS1's effects on ox‐LDL‐dependent cell progression was mediated through regulation of the miR‐320a/LOX1 axis where alterations in cell viability were seen with OIP5‐AS1 knockdown and LOX1 decrease, and partially reversed by miR‐320a depletion.^98^

Another study implicated OIP5‐AS1 in the promotion of ox‐LDL‐mediated endothelial cell apoptosis.^97^ This study found that OIP5‐AS1 was highly expressed in HUVECs supplemented with ox‐LDL, and its silencing resulted in increased cell proliferation due to cell cycle changes, as well as reduced rates of apoptosis. The mechanism of action centred on the recruitment of polycomb repressive complex 2 member, EZH2 to silence GSK‐3β.^97^ The work of Ren et al.^104^ corroborated these findings of increased cell proliferation and decreased apoptosis, along with reduced inflammatory response upon OIP5‐AS1 knockdown. These authors identified miR‐26a‐5p and the AKT/NF‐κβ pathway in the downstream response.

These findings and/or varying mechanisms highlight the need for further study on precise contextual mechanisms for OIP5‐AS1 function. Nevertheless, while there is much left to be understood about the role of OIP5‐AS1 in atherosclerosis, this involvement of OIP5‐AS1 and downstream targets in processes driving atherosclerosis has implications in the development of therapeutics for this leading cause of human vascular disease.

### 3.12 Heart failure

Multiple lncRNA candidates have been linked to heart development and function, as well as various cardiac pathologies.^105^, ^106^, ^107^, ^108^ Stimulated by observations of its enrichment in striated muscle and differentiated cardiomyocytes along with reduced expression in the context of heart failure, a recent study examined roles for OIP5‐AS1 using a model of pressure‐overload induced heart failure (Figure 4).^109^ CRISPR/Cas9 was used to generate knockout mouse models, which revealed a sex‐specific role for OIP5‐AS1, wherein female mice were more prone to heart failure than male mice.^109^ Authors indicated that this sex‐specific difference was tied to changes in cardiomyocyte metabolism in female knockout mice versus scarring and fibrosis, and were also independent of changes in previously identified OIP5‐AS1 modulators such as miR‐7, miR‐29 or HuR.^109^ This cardiac‐health‐related sexual dimorphism has implications in the clinic since there are gender‐related differences in cardiovascular disease for men and women.^110^

### 3.13 Renal transplant rejection

Studies conducted on renal transplant rejection have focused on the correlation between lncRNA expression levels and known cases of rejected or accepted/successful transplants.^111^, ^112^ Such studies have shown that lncRNAs are relevant considerations in detecting transplant rejection.^112^, ^113^, ^114^ OIP5‐AS1 expression levels are inversely correlated to the expression levels of FAS‐AS1 in patients experiencing renal transplant rejection.^112^ FAS‐AS1 is highly expressed in renal transplant rejection and reflects increased lymphocyte activity, which marks the process of acute graft rejection.^112^ Therefore, while not a marker on its own, downregulated expression levels of OIP5‐AS1 in patients that have received renal transplants may be an indicator for the beginnings of acute graft rejection.

### 3.14 Context‐specific roles in cancer: A synopsis

Despite being studied for only a few years, OIP5‐AS1 has already been associated with numerous cancers (Table 2), and meta‐analysis of data on several cancers indicated that dysregulated levels are associated with poor overall survival.^115^ A landmark study from The Cancer Genome Atlas indicate OIP5‐AS1's importance in influencing tumour‐related pathways in varying cancer contexts by modulating a network of prominent cancer regulators including PTEN, ETS1 and PI3K pathway members, unlike other lncRNAs whose function is more tumour specific.^116^, ^117^

Its potential as a tumour suppressor was tested in breast and gynaecological cancer models, based on observations that its locus is deleted or its expression downregulated in basal‐like breast cancer and gynaecological cancers; in these cases, OIP5‐AS1 phenocopied PTEN effects in breast, endometrial and ovarian cancer cells, and broadly regulated other master cancer regulators.^117^

**FIGURE 5:**
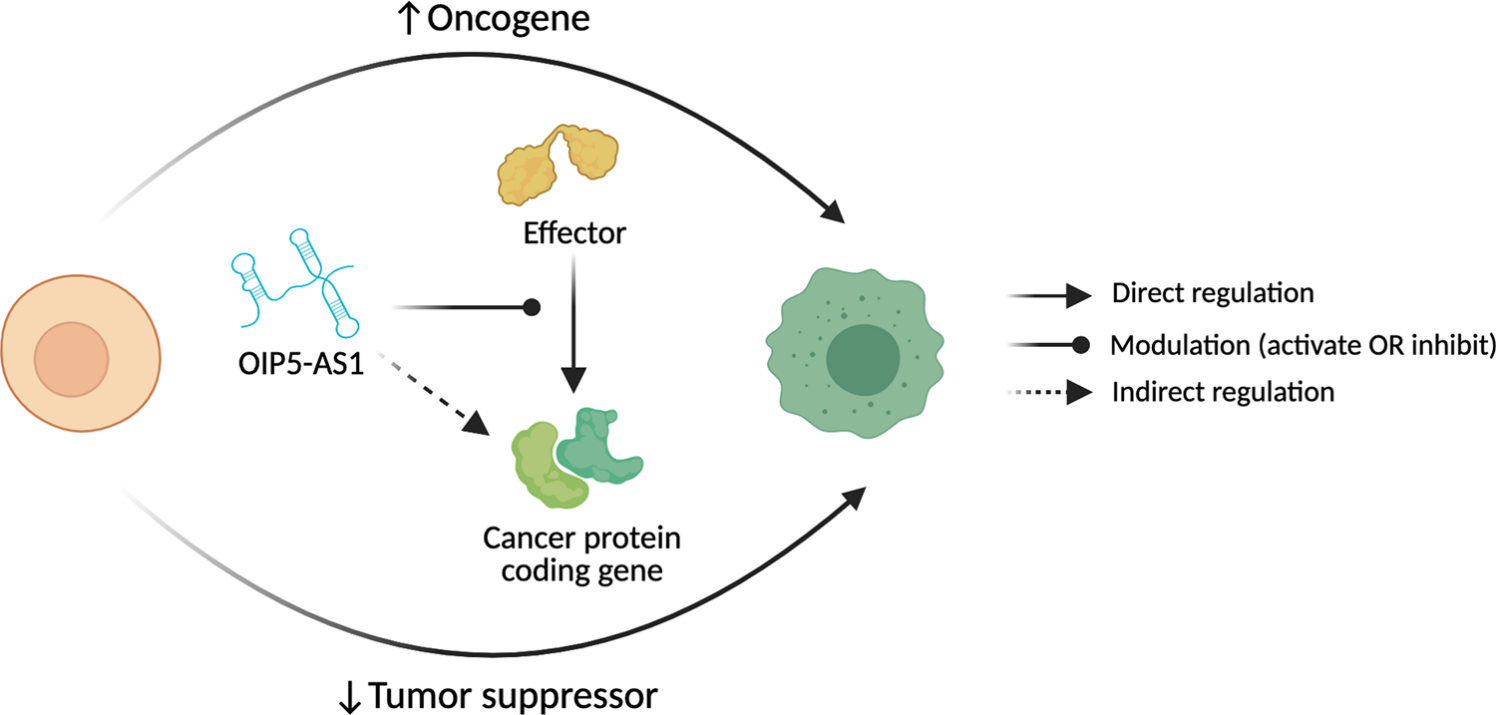
In cancer, OIP5‐AS1 is a master regulator via its actions on effectors of cancer pathways or by mediating cancer‐related factors, with a resultant potential to act as an oncogene or tumour suppressor in a context‐dependent manner

Across a multiplicity of studies, it has been concluded that lncRNAs, including OIP5‐AS1, and their abnormal expression/regulation has a major role in the development and progression of human cancers, as well as drug and treatment resistance.^16^, ^24^, ^49^, ^51^, ^117^, ^118^ This broad influence, and context‐specific function, including functioning as a tumour suppressor or an oncogene in specific tumour types (Figure 5), highlights the need for in‐depth studies to dissect such multifunctional lncRNAs in cancer.

### 3.15 Osteosarcoma: Cisplatin resistance

Cisplatin is a common treatment used in chemotherapy for osteosarcoma^119^, ^120^, ^121^; it mediates DNA destruction as well as gene expression in order to induce tumour cell apoptosis and halt cancer progression. However, an increasing number of osteosarcoma patients have displayed resistance to cisplatin treatment, contributing to low survival rates for osteosarcoma.^122^

Studies on OIP5‐AS1 have examined its role in the development, progression, and treatment regimen for osteosarcoma.^122^, ^123^, ^124^, ^125^ Regarding cisplatin resistance, it has been found to be upregulated in cisplatin‐resistant osteosarcoma cells and tissues, where cells were more susceptible to cisplatin treatment with OIP5‐AS1 knockdown. Cisplatin‐resistant cells developed through the triggering of the P13K/AKT/mTOR pathway via OIP5‐AS1 activity, the depletion of which reduced P13K and AKT signalling, which leads to increased rates of apoptosis. Negative regulation through sponging of miR‐340‐5p reinforces activation of the P13K/AKT/mTOR pathway.^122^

### 3.16 Osteosarcoma: Doxorubicin and radiotherapy resistance

Doxorubicin is also standard chemotherapy for osteosarcoma. OIP5‐AS1 was also upregulated in doxorubicin‐resistant cells in chemo‐resistant patients, and its depletion decreased doxorubicin resistance and tumour cell proliferation, coupled with increased tumour cell apoptosis and better chemotherapy success as measured by tumour weight and volume.^125^

The pathogenesis of OIP5‐AS1‐mediated doxorubicin resistance lies in OIP5‐AS1's binding to miR‐137‐3p. OIP5‐AS1 knockdown resulted in increased miR‐137‐3p and increased cell sensitivity to doxorubicin.^125^

### 3.17 Colorectal cancer

In the context of radiotherapy, it has been found that OIP5‐AS1 affects radiosensitivity via the tumour suppressor DYRK1A; OIP5‐AS1 and DYRK1A are crucial molecular players in the inhibition of cell survival and promotion of apoptosis, leading to increased radiosensitivity in colorectal cancer cells, with the potential to improve outcomes for osteosarcoma via radiotherapy.^24^ Both OIP5‐AS1 and DYRK1A were discovered to be downregulated in radioresistant colorectal cancer cells, and OIP5‐AS1 indirectly upregulates DYRK1A expression via miR‐369‐3p binding and suppression, resulting in the reduction of colorectal cancer cell viability.^24^

### 3.18 Human cervical carcinoma

Since its discovery, much of OIP5‐AS1's role in the regulation of cellular activities including cellular proliferation and apoptosis has been ascribed to its interactions with various forms of RNA. More specifically, it has been found that the up‐ and/or downregulation of OIP5‐AS1 in the context of cellular proliferation, plays a key role in the progression of various diseases including multiple forms of malignant conditions.^115^

One of the key interactions of OIP5‐AS1 in cervical cancer cells was with HuR, which has implications on its stability.^21^ HuR has been found to be abundant in cancers and promotes aspects of tumourigenesis, including reducing the rate of apoptosis, enhancing cell survival, as well as cell proliferation.^126^ The potential for OIP5‐AS1 to affect HuR functions in cancer is due to its sponge functionality which results in HuR being unavailable to bind its target mRNAs.^21^

However, the presence of miR‐424 leads to competition against HuR for binding with OIP5‐AS1; the binding of miR‐424 to OIP5‐AS1 allows for increased concentration of HuR and its availability to bind to its target mRNA, thereby facilitating increased cell proliferation, which is a marked characteristic in malignant conditions.^21^

Through the siRNA‐mediated knockdown of OIP5‐AS1 in HeLa cells, it was also seen that the downregulation or decreased availability of OIP5‐AS1 in HeLa cells correlated with more rapid progression through the cell cycle and enhanced cellular proliferation. Altogether this suggests that reduced OIP5‐AS1 may support progression of human cervical carcinoma.^21^

### 3.19 Multiple myeloma

Multiple myeloma is a haematological malignancy where abnormal expression or dysregulation of miRNAs have been found.^127^, ^128^ OIP5‐AS1 has been implicated in the progression of multiple myeloma and the prognosis for multiple myeloma patients via different mechanisms involving miRNAs.^7^, ^129^

miR‐410 displays increased expression in both newly diagnosed and relapsed multiple myeloma cells, and the detection of high miR‐410 expression has been linked to poor prognosis for multiple myeloma patients.^129^ This is based on miR‐410's role in promoting cell cycle progression and cellular proliferation while inhibiting apoptosis, both of which support tumour growth.^129^

OIP5‐AS1 levels are inversely correlated to miR‐410 expression in multiple myeloma, where miR‐410 levels increase upon OIP5‐AS1 depletion, allowing for the characteristic accumulation of miR‐410 seen in multiple myeloma.^129^ Knockdown of OIP5‐AS1 and the consequent uninhibited function of miR‐410 results in increased cellular proliferation, inhibited apoptosis and overall tumour growth. OIP5‐AS1's regulation of miR‐410 activity contributes to regulation of the KLF10/PTEN/AKT signalling pathway, where PTEN/AKT is downstream of miR‐410 activity, while KLF10 and OIP5‐AS1 action to regulate miR‐410 result in decreased cell proliferation while inducing apoptosis, characterising both as downstream mediators in multiple myeloma progression.^129^

miR‐27a‐3p is an isoform of miR‐27a that has been shown to promote tumourigenesis, proliferation and metastasis in human cancers through its binding to the 3'UTR of target mRNAs.^130^ In the case of the progression of multiple myeloma, miR‐27a specifically binds to the 3'UTR of the mRNA SPRY2 (Sprouty homolog 2).^7^

Recent studies show that miR‐27a‐3p and OIP5‐AS1 expression are inversely correlated.^7^ OIP5‐AS1's effects on cancer characteristics including tumour colony formation, proliferation and cell cycle progression, as well as inducing apoptosis and inhibiting metastasis were explored with the NCI‐H929 and MM1.S cell lines. The interaction between miR‐27a‐3p and OIP5‐AS1 influences these characteristics that mediate multiple myeloma biology. OIP5‐AS1 binding negatively regulates miR‐27a‐3p, and this sponging leads to the upregulation of the protein TSC1, which induces apoptosis and halts characteristics associated with the progression of multiple myeloma.^7^ Supplementation of miR‐27a‐3p in vitro has been found to reverse the effects of OIP5‐AS1 overexpression.^7^

Altogether, expression of OIP5‐AS1, interactions with various miRNAs, and its association with prognostic candidates in multiple myeloma further cements its involvement in various cancers and diseased states.

## 4 CONCLUSION AND FUTURE DIRECTIONS

Evidence mounts that OIP5‐AS1/Cyrano/Oip5os1 has important roles in a variety of cellular processes including regulation of cell proliferation and apoptosis, which translate to a significant impact on the oetiology and progression of numerous diseases (Tables 1 and 2). Primary mechanisms of action depend on intermolecular interactions.^131^ Indeed, OIP5‐AS1's multiplicity of mechanisms for various illnesses ranging from cancers to neurological disorders suggests that there may be unique functions in each system, to result in context‐specific inhibition or promotion of diseased states. One key remaining question pertains to how individual interactions are specified and functionally controlled among numerous possible targets within a cell.

For cancers (Table 2), these processes and activities of OIP5‐AS1 have implications for the monitoring and treatment of malignant conditions. Similarly, for other degenerative and chronic diseases, dissection of these cellular and molecular functions will have significant implications in the clinic, based on its potential as an indicator of the origination, and progression of diseases (Figure 1).^115^ Limitations to current research include many studies being carried out in cell line models, where the spatial context in tumours and other diseased tissues have been lost. Future contextual work modulating OIP5‐AS1 in animal models will provide insight into which contexts its dysregulation is a driver of the particular condition, or which are cases of guilt‐by‐association. Intriguingly, studies to date have shown no overt knockout phenotypes in mouse models,^4^, ^132^ but instead have revealed elegant roles in specific contexts in the heart and brain, for example,^4^, ^109^ suggesting that mechanistic and contextual studies are still needed to fully understand the extent of OIP5‐AS1's functional pleiotropism.

For cancer clinical studies, increased patient numbers will improve confidence in demarcating specific roles for OIP5‐AS1 in each tumour type, especially given the capacity to function as an oncogene or tumour suppressor depending on the context (Table 2). These areas provide direction for future studies on the mechanistics of OIP5‐AS1's function including the unique interactomes that it adopts in each disease context to result in different functional outcomes. Over the long term, more in‐depth studies will be needed to explore if this RNA has realistic potential as a target for new treatment and therapy development.
